# Different HCV Genotype Distributions of HIV-Infected Individuals in Henan and Guangxi, China

**DOI:** 10.1371/journal.pone.0050343

**Published:** 2012-11-30

**Authors:** Di Tian, Lin Li, Yongjian Liu, Hanping Li, Xiaoyuan Xu, Jingyun Li

**Affiliations:** 1 Department of Infectious Disease, Peking University First Hospital, Beijing, China; 2 Department of AIDS Research, State Key Laboratory of Pathogen and Biosecurity, Beijing Institute of Microbiology and Epidemiology, Beijing, China; University of Cincinnati College of Medicine, United States of America

## Abstract

**Background:**

Due to shared transmission routes, hepatitis C virus (HCV) infection is highly prevalent among people infected with human immunodeficiency virus (HIV). Highly active antiretroviral therapy (HAART) is associated with hepatotoxicity, leading to the negative effects on patients with HIV/HCV co-infection. In order to provide valuable information for HCV management in this particular population, we investigated the HCV genotypes in HIV-infected individuals from Henan and Guangxi, the two provinces with the most HIV-infected cases in China.

**Methods:**

Individuals, who acquired HIV infection through various risk routes, were recruited from Henan and Guangxi. Test of antibodies against HCV (anti-HCV) was conducted, and detection of HCV RNA was performed by PCR amplification. HCV subtypes were determined by direct sequencing of amplicons, followed by phylogenetic analysis.

**Results:**

We recruited a total of 1,112 HIV-infected people in this present study. Anti-HCV was detected from 218 (50.1%) patients from Henan and 81 (12.0%) patients from Guangxi, respectively. The highest prevalence of HIV/HCV co-infection was observed from FBDs (former blood donors) (87.2%) in Henan and IDUs (intravenous drug users) (81.8%) in Guangxi, respectively. The seroprevalence rate of HCV among people with sexual contact was significantly higher in Henan than in Guangxi (18.7% vs. 3.5%, *P*<0.05). The positive rate of HCV RNA in Henan and Guangxi was 30.6% (133/435) and 11.2% (76/677), respectively. Moreover, we found that 20 anti-HCV negative samples were HCV positive by PCR amplification. HCV subtype 1b (52.7%) was predominant in Henan, followed by subtype 2a (41.9%). The most frequently detected subtypes in Guangxi were 6a (35.6%) and 3b (32.9%).

**Conclusion:**

The HCV genotype distributions were different in HIV-infected people from Henan and Guangxi. HIV/HCV co-infection was not only linked to the transmission routes, but also associated with the geographic position.

## Introduction

Hepatitis C virus (HCV) is a single, positive-stranded RNA virus, and it belongs to the *Flaviviridae* family [1]. HCV infection is the most important factor causing chronic hepatitis, liver cirrhosis and hepatocellular carcinoma, affecting more than 170 million people worldwide [2]. Due to shared transmission routes and common risk factors, HCV infection is frequently associated with the human immunodeficiency virus (HIV) infection, with a prevalence of approximately 20% to 25% of the HIV-positive population [3]. Since the introduction of highly active antiretroviral therapy (HAART) in the 1990s, the survival rate in HIV-positive individuals has been increasing. Although people with HIV/HCV co-infection have more rapid progression to cirrhosis and hepatocellular carcinoma [3], [4], HCV-induced liver disease is a major cause of morbidity and mortality in HIV-infected people [5]. Several studies have revealed that HAART is associated with an increased risk of antiretroviral-related hepatotoxicity and drug-induced liver injury (DILI) in people with HIV/HCV co-infection [6]. Therefore, it is necessary to screen HCV infection among HIV-infected people [7], especially given a potential treatment for HIV infection.

Although both transmitted through parenteral, sexual and perinatal exposure, HIV and HCV differ in the transmission efficiencies of these routes [8]. Therefore, among HIV-infected individuals, the HCV co-infection rate varies according to the route, through which HIV infection is acquired [9]. Parenteral exposure, such as intravenous drug users (IDUs) or transfusion of blood or blood products, is the most important route for co-infection [10]. Around 90% of HIV-infected IDUs are reported to be HCV co-infected [11]. However, HCV transmission through sexual intercourse appears to be inefficient, and the incidence of HCV infection is less than 15% among HIV-infected people with a sexual risk factor [11]. Knowledge of HCV prevalence in the HIV-infected population is highly valuable for management and treatment of HCV in these particular patients.

**Table 1:** Population characteristics of the study cohort and the seroprevalence rate of HCV among HIV-infected people from various risk groups in Henan and Guangxi.

Infection routes	N		Age (years) [Mean±SD (Range)]		Male gender (%)		Anti-HCV positive (%)	
	Henan	Guangxi	Henan	Guangxi	Henan	Guangxi	Henan	Guangxi
FBDs	187		44.88±7.98 (27–68)		104 (55.6)		163 (87.2)	
Transfusion exposure	33		41.76±15.04 (17–83)		13 (39.4)		19 (57.6)	
Sexual contact	150	512	37.46±9.65 (18–68)	41.50±12.94 (20∼73)	86 (57.3)	335 (65.4)	28 (18.7)	18 (3.5)
IDUs	1	55	25 (25)	36.80±6.53 (27∼54)		47 (85.5)	1 (100)	45 (81.8)
Sexual contact and IDUs		3		35.00±5.20 (29∼38)		2 (66.7)		3 (100)
Perinatal exposure	51		8.96±3.64 (1–17)		30 (58.8)		3 (5.9)	
Unknown	13	107	42.60±17.88 (17–69)	49.29±17.48 (19–81)	8 (61.5)	68 (63.6)	4 (30.8)	15 (14.0)
Total	435	677	37.7±14.4 (1–83)	42.3±13.8 (19–81)	241 (55.4)	452 (66.8)	218 (50.1)	81 (12.0)

**Figure 1:**
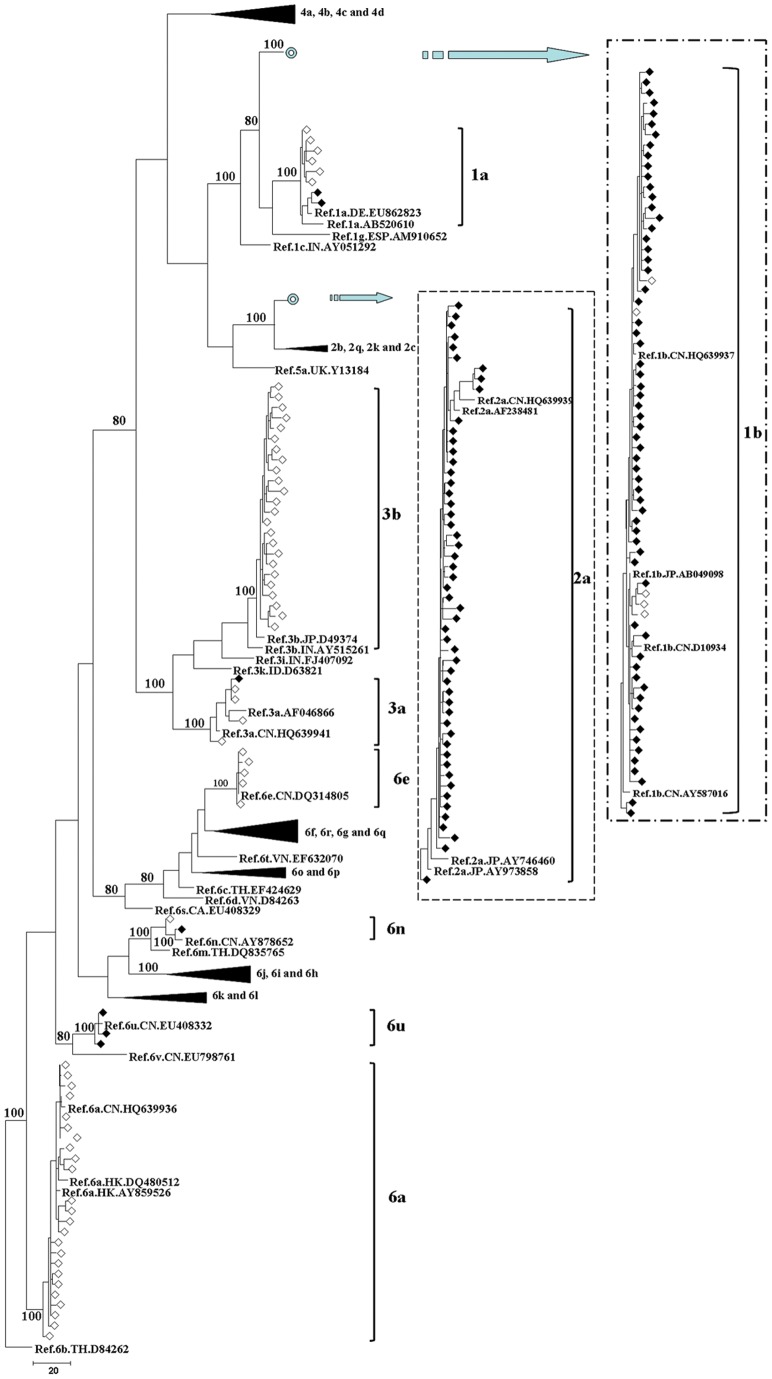
MPT for the HCV NS5B region sequence obtained from HIV/HCV co-infected patients in Henan and Guangxi. The sequences correspond to nucleotides 8,343-9,226 in HCV H77 genome (NC_004102). This dataset included sequences from 242 HCV specimens. The dataset had an aligned length of 884 characters in the dataset, of which, 318 characters are constant, 45 are variable and parsimony-uninformative, and 521 are parsimony-informative. Maximum Parsimony analysis yielded 88 equally parsimonious trees (TL  = 2073, CI  = 0.314, RI  = 0.774, RC  = 0.287, HI  = 0.686). Parsimony bootstrap proportions higher than 70% were indicated along branches. The sequences determined in this study are indicated by the following signs: sequences from Henan (♦), sequences from Guangxi (◊). Reference sequences used to classify HCV genotypes are displayed in bold with the following format: Ref/subtype/country code/accession number.

The HCV genome has a high level of genetic heterogeneity [12]. Previous studies have revealed at least six major genotypes and more than 70 subtypes of this virus [12]. Geographically speaking, HCV genotypes 1, 2 and 3 have a worldwide epidemic, whereas genotype 4 is prevalent in Africa and Middle East. Moreover, genotypes 5 and 6 are found locally in South Africa and Southeast Asia, respectively [13]–[15]. The most commonly detected genotypes in China are 1b and 2a, followed by several less common types. Among the latter ones, subtypes 1a, 3a and 3b have been described among IDUs with HIV-1 co-infection in Yunnan, Guangxi and Xinjiang [16], and subtypes 6a, 6e, 6k, 6n and 6u are observed among IDUs from Jiangsu, Yunnan, Guangxi and Guangdong [17]–[20]. HCV-genotype identification is useful to determine the source of HCV transmission in an infected localized population, and it is clinically important to predict disease outcome and response to anti-HCV therapy. Interestingly, several studies have shown that the risk of hepatotoxicity is much higher in patients with HCV genotype 3 compared with other genotypes when treated with HAART [21], [22]. However, only a few studies have focused on the HCV-genotype distribution in patients with HIV/HCV co-infection through various transmission routes in China [16], [18]–[20], [23]. Further epidemiological studies on HIV/HCV co-infection in China are required in various high-risk areas and groups.

**Table 2:** HCV genotype distributions according to sex, age and HIV infection route in Henan and Guangxi.

			HCV subtypes (%)								
			1a	1b	2a	3a	3b	6a	6e	6n	6u
Henan	Gender	Male	1(1.4)	38(52.0)	32(43.8)					1(1.4)	1(1.4)
		Female	1(1.8)	30(53.6)	22(39.2)	1(1.8)					2(3.6)
	Age (years)	≤20	0	1(33.3)	2(66.7)						
		21–40	1(2.6)	18(47.4)	15(39.5)	1(2.6)					3(7.9)
		41–60	1(1.2)	49(59.8)	31(37.8)					1(1.2)	
		>60			6(100)						
	Infection routes	FBDs		52(54.2)	43(44.8)						1(1.0)
		Transfusion exposure		4(57.1)	3(42.9)						
		Sexual contact	2(10.5)	8(42.1)	6(31.5)	1(5.3)				1(5.3)	1(5.3)
		IDUs									1(100)
		Perinatal exposure		1(50.0)	1(50.0)						
		Unknown		3(75.0)	1(25.0)						
Guangxi	Gender	Male	3(5.0)	5(8.3)	1(1.7)	3(5.0)	20(33.3)	23(38.4)	5(8.3)		
		Female	3(23.1)	1(7.7)		1(7.7)	4(30.7)	3(23.1)		1(7.7)	
	Age (years)	21–40	5(10.8)	2(4.4)	1(2.2)	1(2.2)	15(32.6)	18(39.1)	3(6.5)	1(2.2)	
		41–60	1(4.0)	4(16.0)		2(8.0)	8(32.0)	8(32.0)	2(8.0)		
		>60				1(50.0)	1(50.0)				
	Infection routes	Sexual contact	1(5.9)	4(23.5)	1(5.9)	1(5.9)	4(23.5)	6(35.3)			
		IDUs	1(2.6)	2(5.3)			15(39.5)	14(36.8)	5(13.2)	1(2.6)	
		Sexual contact and IDUs					1(33.3)	2(66.7)			
		Unknown	4(26.7)			3(20.0)	4(26.7)	4(26.7)			

In China, the initial HIV-1 outbreak was caused by IDUs in Yunnan Province in the late 1980s [24], and then HIV was introduced into Guangxi and Xinjiang through heroin trafficking routes [23]. In the early-middle 1990s, unregulated commercial plasma collection was common in the central Chinese provinces, such as Henan, Anhui and Shanxi. The former blood donors (FBDs) in these regions caused the second major epidemic of HIV-1 infection in China [25], constituting a unique cohort related to commercial plasma donation. Unsanitary blood collection among local populations results in HIV and HCV infections [25]–[27]. In order to investigate the incidence of HCV infection and HCV-genotype distribution among HIV-infected people from various risk groups in China, we recruited HIV-infected patients from Henan and Guangxi in the present study. We evaluated the genetic diversity of HCV infection in these two provinces, and identified the regional differences in the HCV-genotype distribution among HIV-infected people. Our findings provided new insights into the management of HIV and HCV transmission and treatment in China.

**Figure 2:**
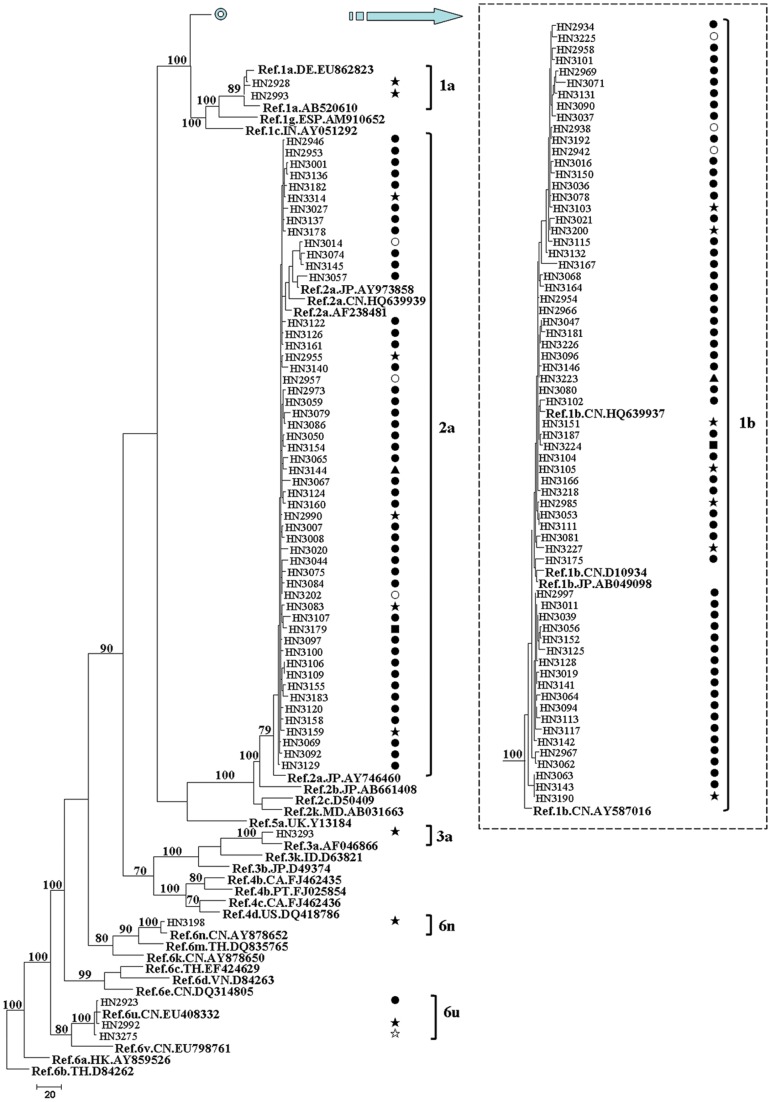
MPT for the HCV NS5B region sequence obtained from HIV/HCV co-infected patients in Henan. The sequences correspond to nucleotides 8343-9226 in HCV H77 genome (NC_004102). This dataset included sequences from 159 HCV specimens. The dataset had an aligned length of 884 characters in the dataset, of which, 335 characters are constant, 43 are variable and parsimony-uninformative, and 506 are parsimony-informative. Maximum Parsimony analysis yielded 88 equally parsimonious trees (TL  = 1945, CI  = 0.276, RI  = 0.859, RC  = 0.237, HI  = 0.724). Parsimony bootstrap proportions higher than 70% were indicated along branches. The HIV transmission route for each patient is listed opposite each sequence: FBDs (•); transfusion exposure (○), sexual contact (★), IDUs (☆), perinatal exposure (▴), unknown infection route (▪). See Figure 1 for reference sequences naming details.

**Figure 3:**
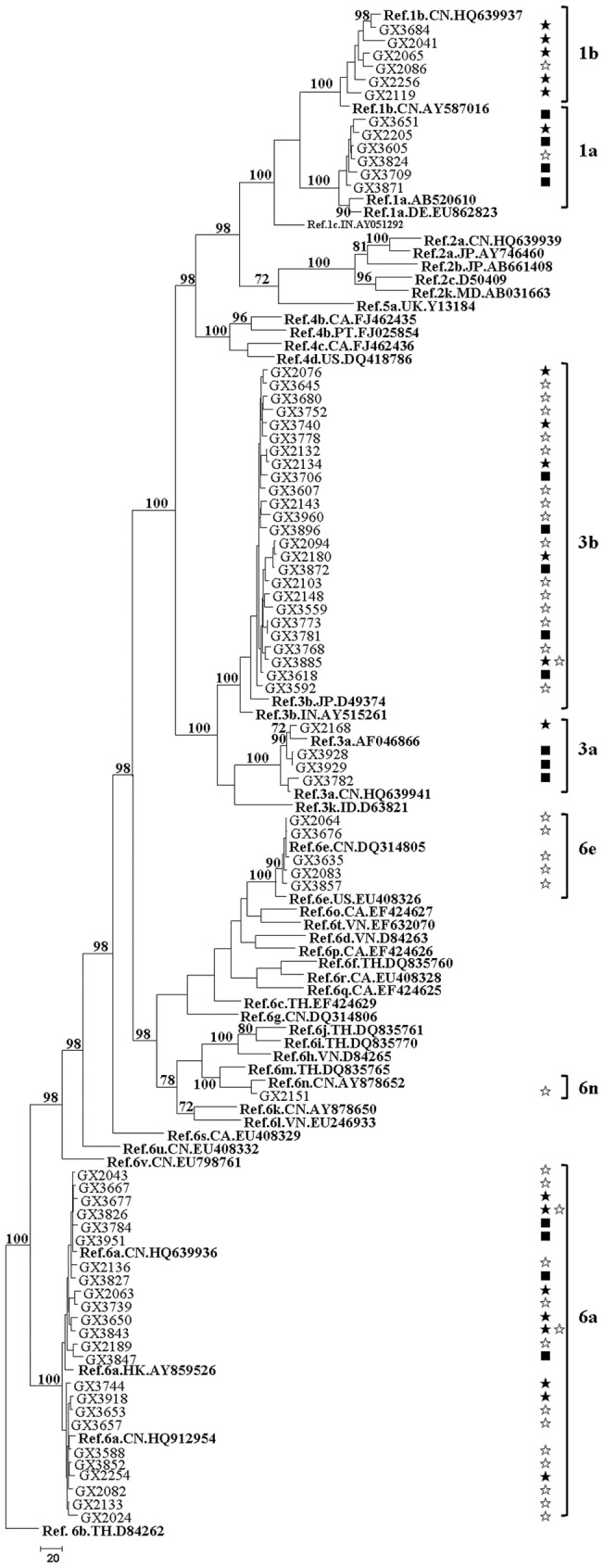
MPT for the HCV NS5B region sequence obtained from HIV/HCV co-infected patients in Guangxi. The sequences correspond to nucleotides 8343-9226 in HCV H77 genome (NC_004102). This dataset included sequences from 116 HCV specimens. The dataset had an aligned length of 884 characters in the dataset, of which, 331 characters are constant, 46 are variable and parsimony-uninformative, and 507 are parsimony-informative. Maximum Parsimony analysis yielded 100 equally parsimonious trees (TL  = 3012, CI  = 0.233, RI  = 0.678, RC  = 0.282, HI  = 0.767). Parsimony bootstrap proportions higher than 70% were indicated along branches. See Figure 1 and Figure 2 for reference sequences naming details and signing scheme.

## Results

### Prevalence of HCV infection among HIV-infected people in Henan and Guangxi

We recruited a total of 1,112 HIV-infected individuals in the provinces of Henan and Guangxi, China. The blood samples obtained in Henan were collected from various risk groups, including 187 FBDs, 33 individuals with transfusion exposure, 150 individuals with a sexual risk (nine homosexuals and 141 heterosexuals), one IDU, 51 individuals with perinatal exposure and 13 individuals with an unknown infection route. Subjects from Guangxi included 512 individuals with a sexual risk (15 homosexuals and 497 heterosexuals), 55 IDUs, three individuals with both sexual and intravenous drug use risks, and 107 individuals with an unknown infection route. Among all the participants, 693 (62.3%) were male and 419 (37.7%) were female, with a median age of 40.5±14.2 years (range 1–83 years). Table 1 lists the age range for each patient group. The overall seroprevalence rate of HCV in HIV-infected subjects was 26.9% (299/1,112). Among these subjects, anti-HCV was detected in 218 (50.1%) patients from Henan and 81 (12.0%) from Guangxi. For various risk groups, the highest prevalence of HIV/HCV co-infection was observed in FBDs (87.2%), followed by IDUs (82.1%) and people who acquired HIV-infection via transfusion (57.6%). HIV/HCV co-infection was relatively rare (5.9%) in the group of people who acquired HIV-infection through perinatal exposure. The seroprevalence rate of HCV among people with sexual contact was significantly higher in Henan than in Guangxi (18.7% vs. 3.5%, *P*<0.05). Table 1 shows the positive rate of anti-HCV among various risk groups in Henan and Guangxi. Due to the poor health condition in some of the HIV-infected regions, CD4 lymphocyte count was not available for all subjects. Among the 1,112 HIV-infected individuals, 396 (35.6%) subjects were tested for CD4 lymphocyte count (242 subjects from Henan and 154 subjects from Guangxi), with a median CD4 cell count of 346±249 cells/mm^3^.

**Table 3:** Primers used in this study.

Region	Nested PCR	Name	Sequences(5′–3′)	Size (bp)	Position(H77)
5′NCR/C	1^st^ PCR	NCR-18F	GGCGACACTCCRCCATRRATCACT		18–41
		NCR-1293R*	CCARTTYAKCATCATRTCCCANGCCAT		1319–1293R
	2^nd^ PCR	NCR-35F*	RATCACTCCCCTGTGAGGAACTWCTGT		35–61
		NCR-849R	ARGAAGATAGARAARGAGCAACCRGG	840	874–849R
C/E2	1^st^ PCR	C/E2-732F	GCCGACCTCATGGGRTAYAT		732–751
		C/E2-2187R	ARTTBTYDGTRCANGGRTARTGCCA		2211–2187R
	2^nd^ PCR	C/E2-87F1	CCYGGTTGCTCYTTYTCTATCTT		849–871
		C/E2-2130R*	GTNADCCARGGHCCNGMNCCRCA	1322–1346	2152–2130R
NS5B	1^st^ PCR	NS5B-8247F	GGSTTYTCGTATGAYACCMGBTGYTTTGA		8247–8275
		NS5B-9325R	CTACCCCTACRGSRAGYAGGAGTAGGC		9351–9325R
	2^nd^ PCR	NS5B-8266F*	GCTGYTTTGAYTCAACNGTCAC		8266–8287
		NS5B-9276R*	GRGCMYGRGACACGCTGTGATASATGTC	1037	9303–9276R

* Primers used for sequencing.

**Table 4:** PCR reaction used in this study.

Region	Nested PCR	Reaction program
**5′NCR/C**	1^st^ PCR	50°C for 30 min, 94°C for 1 min, 3 PCR cycles of 94°C for 1 min, 50°C for 1 min, and 72°C for 2 min, 32 PCR cycles of 94°C for 15 sec, 50°C for 30 sec, and 72°C for 1.5 min; with a final extension at 72°C for 10 min.
	2^nd^ PCR	94°C for 1 min, 3 PCR cycles of 94°C for 1 min, 55°C for 1 min,and 72°C for 1.5 min, 32 PCR cycles of 94°C for 15 sec, 55°C for 30 sec, and 72°C for 50 sec, with a final extension at 72°C for 10 min.
**C/E2**	1^st^ PCR	50°C for 30 min, 94°C for 1 min, 3 PCR cycles of 94°C for 1 min, 50°C for 1min, and 72°C for 2 min; 32 PCR cycles of 94°C for 15 sec, 50°C for 30 sec, and 72°C for 1.5min, with a final extension at 72°C for 10min.
	2^nd^ PCR	94°C for 1 min, 3 PCR cycles of 94°C for 1 min, 55°C for 1 min, and 72°C for 2 min; 32 PCR cycles of 94°C for 15 sec, 55°C for 30 sec, and 72°C for 80 sec; with a final extension at 72°C for 10 min.
**NS5B**	1^st^ PCR	50°C for 30 min, 94°C for 1 min, 3 PCR cycles of 94°C for 1 min, 50°C for 1 min, and 72°C for 2 min; 32 PCR cycles of 94°C for 15 sec, 50°C for 30 sec, and 72°C for 1.5 min; with a final extension at 72°C for 10 min.
	2^nd^ PCR	94°C for 1 min; 3 PCR cycles of 94°C for 1 min, 55°C for 1 min, and 72°C for 2 min; 32 PCR cycles of 94°C for 15 sec, 55°C for 30 sec, and 72°C for 80 sec; with a final extension at 72°C for 10 min.

### Amplification of HCV 5′NCR/C, C/E2 and NS5B regions

The approximate lower limits of detection of the PCR amplification were 150 copies/mL for 5′NCR/C region, 300 copies/mL for C/E2 region and 2,400 copies/mL for NS5B regions. We performed PCR amplification on all the 1,112 HIV-positive samples for 5′NCR/C, C/E2 and NS5B regions. A sample was regarded as HCV RNA positive once the PCR amplification was positive in one of the three regions. PCR amplification was performed in duplicate if the sample was not positive for all the three regions. Among these samples, we successfully amplified 191 5′NCR/C fragments, 197 C/E2 fragments and 200 NS5B fragments. As a result, 110 anti-HCV positive samples were negative for HCV PCR amplification with all the three regions. The negative rate for HCV PCR amplification in anti-HCV positive patients was high among patients who acquired HIV-infection through perinatal exposure or transfusion (66.7% and 63.2%, respectively), intermediate among patients who acquired HIV-infection through sexual contact and FBDs (41.3% and 39.9%, respectively), and low among IDUs (20.0%). In contrast, 20 anti-HCV negative samples were positive for HCV PCR amplification, of which eight samples were positive for HCV RNA with all the three regions, three samples were positive for HCV RNA with two regions, and nine samples were positive for HCV RNA with one region. However, as for CD4 cell count, there was no significant difference between those with anti-HCV positive and those with anti-HCV negative but positive HCV RNA (384±259 cells/mm^3^ vs. 334±136 cells/mm^3^, p = 0.617). The proportion of patients tested for CD4 cell count in both groups were relatively low (57.4% and 35%) because of poor health conditions. Hence, among the 1,112 HIV-infected patients, a total of 209 (18.8%) were positive for HCV RNA. HCV-RNA positive rate was 30.6% (133/435) and 11.2% (76/677) in Henan and Guangxi, respectively.

PCR amplification was performed in duplicate if the amplicon was not successfully sequenced. The sequencing failure was possibly caused by heterozygosity of the HCV genome. In total, 187 5′NCR/C amplicons, 190 C/E2 amplicons and 197 NS5B amplicons were successfully sequenced. Among these amplicons, 173 contained the sequences of all the three regions, and 35 contained one or two sequences of the three regions.

### HCV-subtype distribution in the study population

In order to study the HCV-subtype distribution, we constructed three phylogenetic trees based on the sequences of 5′NCR/C, C/E2 and NS5B fragments, respectively. They corresponded to the nucleotides 91-681, 915-1,835 and 8,343-9,226 in the numbering of H77 genome (NC_004102), respectively. Figure 1 shows that we identified nine HCV subtypes, including 1a, 1b, 2a, 3a, 3b, 6a, 6e, 6n and 6u, according to the phylogenetic analysis of NS5B fragments. The same subtype pattern was detected by the sequence analysis of 5′NCR/C and C/E2 fragments (Figure S1 and Figure S2). Moreover, 173 samples were genotyped by all the three regions, 20 samples were genotyped by two regions, and 15 samples were genotyped by only one region. Among the 193 samples genotyped by two or three regions, we obtained a total of six discordant results, suggesting the existence of mixed-genotype infection or recombination in these six patients.

Table 2 demonstrates the HCV subtype distribution by gender, age and HIV infection routes. Most genotyped samples were male in Guangxi, while only half of the genotyped samples were male in Henan (82.2% vs. 56.6%, p<0.01). HCV subtype was determined mainly in individuals between 41–60 years (82/129, 63.6%) from Henan and in individuals between 21–40 years (46/73, 63.0%) from Guangxi. HCV subtypes could be classified into 1a, 1b, 2a, 3a, 6n and 6u by sequence analysis in Henan. HCV subtype 1b (54.2%) was predominant among FBDs, followed by subtype 2a (44.8%). Only one FBD was classified as subtype 6u. The most frequently identified subtypes among IDUs from Guangxi were 3b (39.5%) and 6a (36.8%), and several other subtypes were also identified. Six subtypes were identified among the patients with sexual contact in Guangxi. Subtype 6a was the most prevalent, with an incidence of 35.3% among these patients. Subtypes 3b and 1b were the second most prevalent genotypes, with an incidence of 23.5% respectively. The least prevalent subtypes were 1a (5.9%), 2a (5.9%) and 3a (5.9%). On the other hand, subtypes 1b and 2a accounted for 42.1% and 31.5% of all the patients with sexual contact in Henan, respectively. Surprisingly, subtypes 3b and 6a were completely absent from all the patients with sexual contact in Henan, genotype 2 was almost absent in Guangxi, and subtype 2a was detected in only one patient with sexual contact.

In order to investigate the HCV-subtype distribution among HIV-infected people through various transmission routes in Henan and Guangxi, we constructed the Maximum Parsimonious Tree (MPT) (Figure 2 and Figure 3) with the NS5B region sequences. The same subtype pattern was detected by the sequence analysis of 5′NCR/C and C/E2 fragments (Figure S3, Figure S4, Figure S5 and Figure S6). Figure 2 shows that NS5B region sequences from Henan were grouped into six clades, and they were labeled as 1a, 1b, 2a, 3a, 6n and 6u containing two, 66, 53, one, one and three sequences, respectively. Among the 126 patients with the available NS5B region sequences, 119 (94.4%) patients were grouped into clade 1b or 2a. Sequences in these two clades were obtained from various transmission routes, and the vast majority in these clades was FBDs. All the sequences in clades 1a, 3a and 6n were obtained from the patients with sexual contact. Although transmission route was similar, the phylogenetic analysis revealed a complex HCV-subtype distribution in Guangxi. Among the 71 NS5B region sequences from Guangxi, six, six, four, 25, 24, five and one sequences were grouped into clades 1a, 1b, 3a, 3b, 6a, 6e and 6n, respectively (Figure 3). Clades 6e and 6n were prevalent only in IDUs, while sequences in clade 3a were not from IDUs. Clades 1a, 1b, 3b and 6a were prevalent not only in IDUs, but also in the patients with sexual contact.

## Discussion

In China, the reported incidence rate of HCV infection is low (<1%) in the general populations [23], [28]. Since HIV and HCV have similar transmission routes, the incidence rate of HCV/HIV co-infection is apparently high among HIV-infected people, regardless of the route of HIV acquisition. In the present study, we found that the seroprevalence rate of HCV in HIV-infected people was 26.9% (299/1,112). This prevalence was significantly higher than those reported data in the general populations. However, it was lower than the reported data (57%) in a previous study [16]. The prevalence of HCV infection varies with the mode of HIV transmission [29]. Between 1992 and 1995, uncontrolled blood collection from FBDs in the central Chinese provinces of Henan, Anhui and Shanxi caused the second major epidemic in China [25]. The HIV/HCV co-infection rate (163/187, 87.2%) among FBDs observed in this study was higher than that reported by Zhang et al. (82.2%) and lower than that reported by Shang et al. (96.6%) [16], [23]. Although paid blood donation is the predominant mode of HIV transmission in Henan, HIV infection is frequently detected in cases of transfusion, sexual contact and perinatal exposure. We detected a high prevalence of HIV/HCV co-infection (57.6%) among people with HIV infection through transfusion in Henan. A high HCV prevalence of 18.7% was also observed in individuals who acquired HIV infection through sexual contact. Our results were consistent with the published data, suggesting that HCV transmission through sexual contact is efficient [23]. Our study also indicated that HCV infection was relatively rare through perinatal exposure, with a prevalence of 5.9% for HIV/HCV co-infection. A recent meta-analysis demonstrated that the risk of vertical transmission of HCV is enhanced by HIV co-infection [30]. To better understand the effect of HIV infection on HCV vertical transmission, large-scale investigations should be performed among mothers with HIV/HCV co-infection or HCV mono-infection. The HCV prevalence in HIV-positive people in Guangxi showed a large variation, ranging from 3.5% in people with sexual contact to 81.8% in IDUs. Interestingly, the proportion of HIV-infected people with sexual contact who were anti-HCV positive was lower in Guangxi than in Henan (18.7% vs. 3.5%, P<0.05), suggesting that the HIV/HCV co-infection rate was not only related to the transmission routes, but also associated with the geographic position.

HCV infection can be serologically confirmed through the anti-HCV detection. However, a positive anti-HCV test cannot distinguish the majority of patients with chronic infection from a minority of those who have spontaneously cleared the virus [5]. Several factors are associated with spontaneous resolution of HCV viremia, including host factors such as age, sex, ethnicity and immune response [31], [32]. The rate of spontaneous HCV resolution can range from 10% to 60% [33]. In our study, even samples with HCV viral load as low as 150 copies/mL could be successfully amplified. As a result, 63.2% (189/299) of anti-HCV positive patients were positive for HCV RNA. This was in line with a previous report, in which 72 out of 107 anti-HCV positive blood samples (67.3%) were positive for HCV RNA [2], but this result was lower than a recent study (71.4%) [20]. It is more likely that some HCV RNA-negative patients had spontaneously controlled HCV replication before HIV infection, and the others were due to low viral titers. This may also be related to the selected population, as anti-HCV false-positive results are more likely to occur among the populations with a low HCV prevalence [7]. In our study, the negative rate of HCV PCR amplification among anti-HCV positive patients showed a large variation among different patient groups: about 20.0% in IDUs, about 40% in people who acquired HIV infection through sexual contact and FBDs, and above 60% in people who acquired HIV infection through perinatal exposure or transfusion. We hypothesized that those without HCV infection in the last group were more likely to obtain anti-HCV from their mothers or blood donors. This deserves further investigation to examine the relationship between anti-HCV and HCV RNA status in people with distinct routes of HIV transmission. In immunocompromised people, especially in HIV/HCV co-infected people, HCV infection can be present despite a negative anti-HCV test [5]. In this study, we confirmed that 20 anti-HCV negative patients had the HCV infection by PCR amplification. Therefore, qualitative HCV-RNA testing is recommended in the diagnosis of HCV infection in this population.

HCV genotype appears to be an important predictor of clinical progression of HCV infection as well as patient response to antiviral therapies [34]. The gold standard for HCV genotyping is direct sequencing of PCR products followed by phylogenetic analysis (compare with the consensus sequences in GenBank or the Los Alamos HCV database) [12], [35]. Our study was designed with the sequences of 5′NCR/C, C/E2 and NS5B regions for HCV genotyping, revealing a high level of concordance among the three regions. HCV subtypes determined by two or three genetic regions were not in agreement for six of the 193 (3.1%) specimens. Complete genome sequences may be able to distinct these recombinants from superinfection. As recombination remains an infrequent event, routine genotyping in more than one subgenomic region for clinical use is not warranted [35].

In the present study, we identified six HCV subtypes in HIV-infected people in Henan, including 1a, 1b, 2a, 3a, 6n and 6u (Figure 1). These results were not consistent with some previous studies [16], [23], in which only HCV subtypes 1b and 2a are detected. It is probably because subjects in these two studies are only from FBDs and their sexual partners. The circulating strains of HCV in Guangxi showed a large phylogenetic diversity, with a total of eight subtypes (subtypes 1a, 1b, 2a, 3a, 3b, 6a, 6e and 6n) (Figure 1). In IDUs from Guangxi, 3b was the major HCV subtype (39.5%), which was followed by 6a (36.8%), 6e (13.2%), 1b (5.3%), 1a (2.6%), and 6n (2.6%). Although Guangxi is the neighbor of Guangdong Province, subtype 3a was absent from IDUs in Guangxi, which was the second most prevalent subtype in IDUs from Guangdong [20]. In our study, the HCV-genotype distribution in Henan was distinct from that observed in Guangxi (Figure 2 and Figure 3). Subtype 1b (52.7%) was the most predominant in Henan, followed by subtype 2a (41.9%), which was consistent with other reports in general populations and FBDs in China [16], [23], [36]. In Henan, this pattern was observed not only among FBDs but also among people who acquired HIV infection from other routes, such as transfusion, sexual contact and perinatal transmission. Although the prevalence of genotype 6 was very low (3.1%) in Henan, it was more prevalent in Guangxi (43.8%). There are two possible interpretations for the observed difference. One is that the leading cause of HIV infection in Henan was the blood donation, while IDU or sexual contact was the main route of HIV transmission in Guangxi. The other interpretation is that the size of HIV/HCV co-infected patients in Guangxi was relatively small in the current study. Interestingly, we found a distinct HCV-genotype distribution among people who acquired HIV infection through sexual contact in Henan and Guangxi. Subtypes 6a and 3b were absent from people with sexual contact in Henan, but they accounted for 35.3% and 23.5% of HCV infection in Guangxi, respectively. It suggested that HCV-genotype distribution is not only associated with the transmission route of HIV,but also associated with the geographic position.

## Conclusions

Taken together, our study revealed that the HCV genotype distributions in HIV-infected people between Henan and Guangxi are different. In Henan, HIV/HCV co-infection is frequent in FBDs and people who acquired HIV infection through transfusion or sex contact. HCV subtypes 1b and 2a appear to be the predominant subtypes in HIV infection from various transmission routes. However, HIV/HCV co-infection is mainly associated with intravenous drug use in Guangxi, and the predominant HCV subtypes are 3b and 6a. These results also suggested that HIV/HCV co-infection is not only linked to the transmission mode, but also associated with geographic position. Due to the high HCV prevalence among HIV-infected patients and hepatotoxicity of HAART, systematic HCV screening becomes an important consideration when an antiretroviral therapy is given [37].

## Materials and Methods

### Study subjects and serological testing

A total of 1,112 HIV-1 positive individuals were recruited in this study from Henan and Guangxi between 2009 and 2011 in the local Center for Disease Control and Prevention. Patients who had been submitted to an antiviral therapy for HCV were excluded. Patients acquired HIV infection from various risk routes as follows, paid blood donation, transfusion of blood or blood products, sexual contact (homosexuals and heterosexuals), intravenous drug injection, perinatal exposure and other unknown infection routes. Diagnosis of HCV infection was established by a positive test for anti-HCV. This study was approved by the ethical committee of the Peking University People's Hospital, and all participants were provided written consent.

Whole blood samples were collected. Plasma and peripheral blood mononuclear cells were obtained by Ficoll-hypaque density gradient centrifugation and stored at −80°C. HIV antibodies (anti-HIV-1 and anti-HIV-2) were detected by enzyme-linked immunosorbent assay (ELISA) and then further confirmed by Western blot in the local Center for Disease Control and Prevention. Plasma samples were screened for anti-HCV using an enzyme immunoassay kit (Livzon Diagnostics Inc., Zhuhai, China) following the manufacturer's instructions. Each experiment was performed in duplicate.

### RNA extraction and amplification of subgenomic-regions

Viral RNA was extracted from 200 ìL of plasma with a High Pure Viral RNA Kit in accordance with the manufacturer's instructions (Roche Applied Science, Mannheim, Germany). Purified RNA was used as the template for reverse transcription PCR (RT-PCR). In order to examine the genetic diversity and HCV genotypes, a 5′NCR/C, a C/E2 region and a NS5B region were selected. Primer pairs used in the nested PCR were designed or modified from the previous reports [17], [18], [36]. Table 3 lists the full details of primers used in our study. To determine the sensitivity of the region-specific PCR assays, we performed serial dilutions of samples with known HCV RNA levels from different genotypes and performed each of the three PCR amplifications to calculate the lower limit of detection. The first PCR reaction was performed using One Step RNA PCR Kit (AMV) (TaKaRa, Dalian, China). The second PCR reaction was performed using Premix Taq Kit (TaKaRa, EX Taq^TM^ Ver) (TaKaRa, Dalian, China). Table 4 lists the detailed conditions of PCR reaction used in our study. Amplocons were separated on 1% agarose gel stained with ethidium bromide and then sequenced from both directions in Beijing Genomics Institute. All the three regions were amplified for both anti-HCV positive samples and anti-HCV negative samples. For those samples with negative amplification for one or two of the three regions, PCR amplification was performed in duplicate.

### HCV genotyping and phylogenetic analysis

The obtained original sequences were assembled with ContigExpress (VectorNTI Suite 8.0; Invitrogen, USA) and aligned with HCV reference sequences using Bioedit and ClustalX. Alignment was manually adjusted to maximize alignment and minimize gaps. Clear sequence information for 591nt of 5′NCR/C region, 921nt of the C/E2 region, and 884nt of NS5B region was selected for genotyping and phylogenetic analysis. HCV genotypes were assigned following the phylogenetic analysis, in which the sequences were compared with reference sequences from the HCV database located in Loa Alamos (http://hcv.lanl.gov/content/sequence/HCV/ToolsOutline.html). The tree construction procedure was performed in PAUP* version 4.0b10. All characters were equally weighted, and gaps were treated as missing data. Trees were inferred using the heuristic search option with tree bisection-reconnection (TBR) branch swapping and 1,000 random sequence additions. Max-trees were set to 5,000, branches of zero length were collapsed, and all parsimonious trees were saved. The reliability of trees was tested by non-parametric bootstrapping in 1,000 replications. Cluster bootstrap values above 70% were considered as significant [12]. Descriptive tree statistics, including tree length (TL), consistency index (CI), retention index (RI), rescaled consistency index (RC) and homoplasy index (HI), were calculated for each obtained MPT.

### Statistical analysis

Statistical analysis was carried out using SPSS software version 13.0 (SPSS, Chicago, IL, USA). Data were expressed as means ± SD. Normality of the variables was examined using Kolmogorov-Smirnov test. Student's t test was used to evaluate the differences in non-categorical variables, whereas chi square test or Fisher's exact test was used for categorical variables. All tests were two-sided, and a *p*-value of less than 0.05 was considered as statistically significant.

### Nucleotide sequence accession numbers

The nucleotide sequences generated in this study have been submitted to the GenBank nucleotide sequence database. The accession numbers are JX960977 to JX961173, JX961174 to JX961363, and JX961364 to JX961550.

## Supporting Information

Figure S1 **MPT for the HCV 5′NCR/C region sequence obtained from HIV/HCV co-infected patients in Henan and Guangxi.** The sequences correspond to nucleotides 91–681 in HCV H77 genome (NC_004102). This dataset included sequences from 235 HCV specimens. The dataset had an aligned length of 594 characters in the dataset, of which, 354 characters are constant, 67 are variable and parsimony-uninformative, and 173 are parsimony-informative. Maximum Parsimony analysis yielded 58 equally parsimonious trees (TL  = 941, CI  = 0.222, RI  = 0.817, RC  = 0.182, HI  = 0.778). Parsimony bootstrap proportions higher than 70% were indicated along branches. See Figure 1 for reference sequences naming details and signing scheme.
(TIF)

Figure S2 **MPT for the HCV C/E2 region sequence obtained from HIV/HCV co-infected patients in Henan and Guangxi.** The sequences correspond to nucleotides 915-1,835 in HCV H77 genome (NC_004102). This dataset included sequences from 241 HCV specimens. The dataset had an aligned length of 945 characters in the dataset, of which, 200 characters are constant, 70 are variable and parsimony-uninformative, and 675 are parsimony-informative. Maximum Parsimony analysis yielded 100 equally parsimonious trees (TL  = 2911, CI  = 0.175, RI  = 0.823, RC  = 0.144, HI  = 0.825). Parsimony bootstrap proportions higher than 70% were indicated along branches. See Figure 1 for reference sequences naming details and signing scheme.
(TIF)

Figure S3 **MPT for the HCV 5′NCR region sequence obtained from HIV/HCV co-infected patients in Henan.** The sequences correspond to nucleotides 91-681 in HCV H77 genome (NC_004102). This dataset included sequences from 156 HCV specimens. The dataset had an aligned length of 594 characters in the dataset, of which, 373 characters are constant, 60 are variable and parsimony-uninformative, and 161 are parsimony-informative. Maximum Parsimony analysis yielded 9 equally parsimonious trees (TL  = 727, CI  = 0.305, RI  = 0.838, RC  = 0.256, HI  = 0.695). Parsimony bootstrap proportions higher than 70% were indicated along branches. See Figure 1 and Figure 2 for reference sequences naming details and signing scheme.
(TIF)

Figure S4 **MPT for the HCV 5′NCR region sequence obtained from HIV/HCV co-infected patients in Guangxi.** The sequences correspond to nucleotides 91-681 in HCV H77 genome (NC_004102). This dataset included sequences from 109 HCV specimens. The dataset had an aligned length of 594 characters in the dataset, of which, 363 characters are constant, 71 are variable and parsimony-uninformative, and 160 are parsimony-informative. Maximum Parsimony analysis yielded 25 equally parsimonious trees (TL  = 924, CI  = 0.289, RI  = 0.774, RC  = 0.224, HI  = 0.711). Parsimony bootstrap proportions higher than 70% were indicated along branches. See Figure 1 and Figure 2 for reference sequences naming details and signing scheme.
(TIF)

Figure S5 **MPT for the HCV C/E2 region sequence obtained from HIV/HCV co-infected patients in Henan.** The sequences correspond to nucleotides 915-1,835 in HCV H77 genome (NC_004102). This dataset included sequences from 159 HCV specimens. The dataset had an aligned length of 945 characters in the dataset, of which, 221 characters are constant, 65 are variable and parsimony-uninformative, and 659 are parsimony-informative. Maximum Parsimony analysis yielded 28 equally parsimonious trees (TL  = 2546, CI  = 0.231, RI  = 0.816, RC  = 0.188, HI  = 0.769). Parsimony bootstrap proportions higher than 70% were indicated along branches. See Figure 1 and Figure 2 for reference sequences naming details and signing scheme.
(TIF)

Figure S6 **MPT for the HCV C/E2 region sequence obtained from HIV/HCV co-infected patients in Guangxi.** The sequences correspond to nucleotides 915-1,835 in HCV H77 genome (NC_004102). This dataset included sequences from 109 HCV specimens. The dataset had an aligned length of 935 characters in the dataset, of which, 217 characters are constant, 51 are variable and parsimony-uninformative, and 667 are parsimony-informative. Maximum Parsimony analysis yielded 28 equally parsimonious trees (TL  = 4759, CI  = 0.191, RI  = 0.693, RC  = 0.132, HI  = 0.809). Parsimony bootstrap proportions higher than 70% were indicated along branches. See Figure 1 and Figure 2 for reference sequences naming details and signing scheme.
(TIF)
