# Supplementary figures and images for: Different HCV Genotype Distributions of HIV-Infected Individuals in Henan and Guangxi, China
Source: PLoS One. 2012 Nov 30;7(11):e50343. doi: 10.1371/journal.pone.0050343 (PMC3511438; doi:10.1371/journal.pone.0050343)

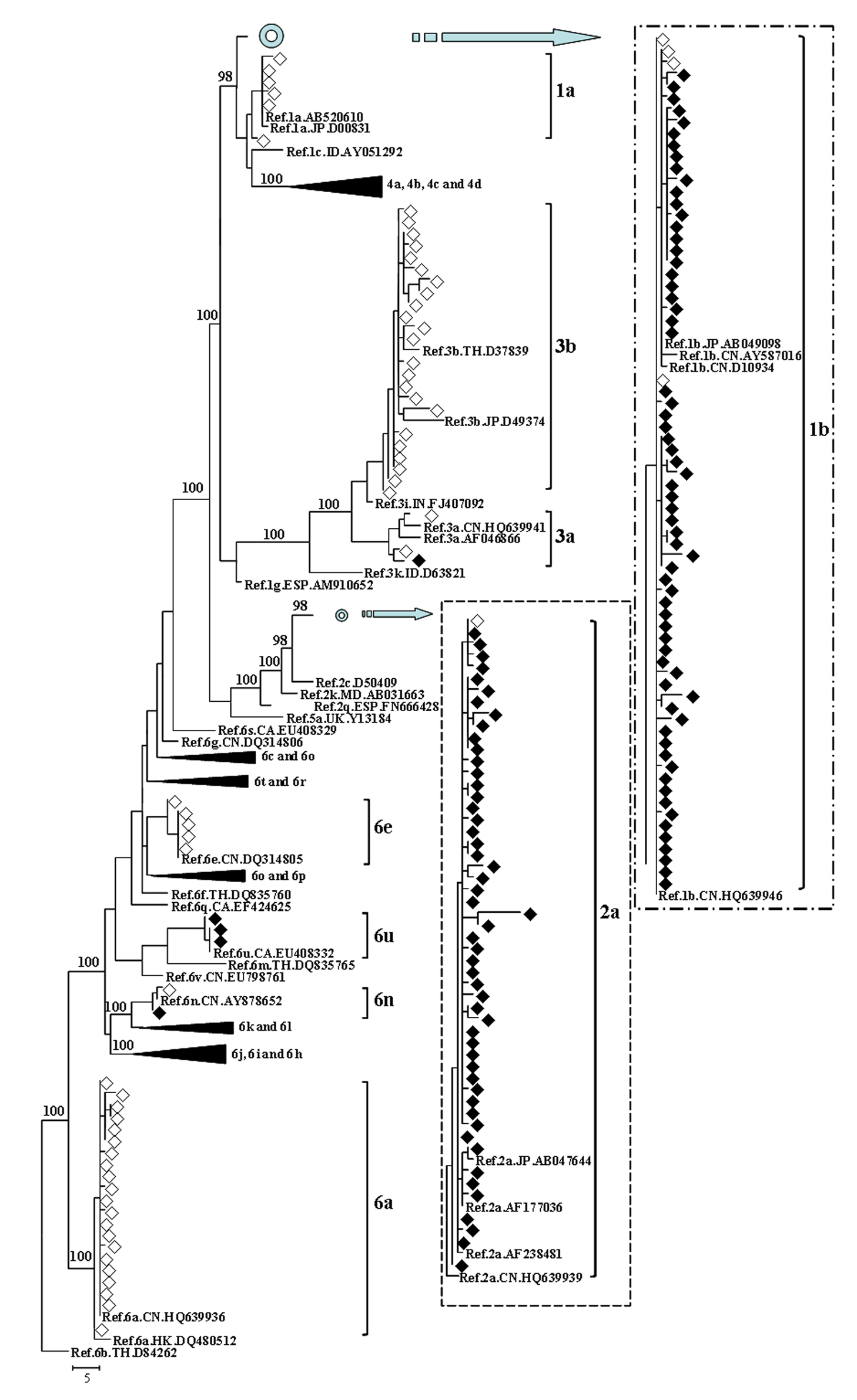

Supplement: Figure S1 — MPT for the HCV 5′NCR/C region sequence obtained from HIV/HCV co-infected patients in Henan and Guangxi. The sequences correspond to nucleotides 91–681 in HCV H77 genome (NC_004102). This dataset included sequences from 235 HCV specimens. The dataset had an aligned length of 594 characters in the dataset, of which, 354 characters are constant, 67 are variable and parsimony-uninformative, and 173 are parsimony-informative. Maximum Parsimony analysis yielded 58 equally parsimonious trees (TL = 941, CI = 0.222, RI = 0.817, RC = 0.182, HI = 0.778). Parsimony bootstrap proportions higher than 70% were indicated along branches. See Figure 1 for reference sequences naming details and signing scheme. (TIF) [file pone.0050343.s001.tif]

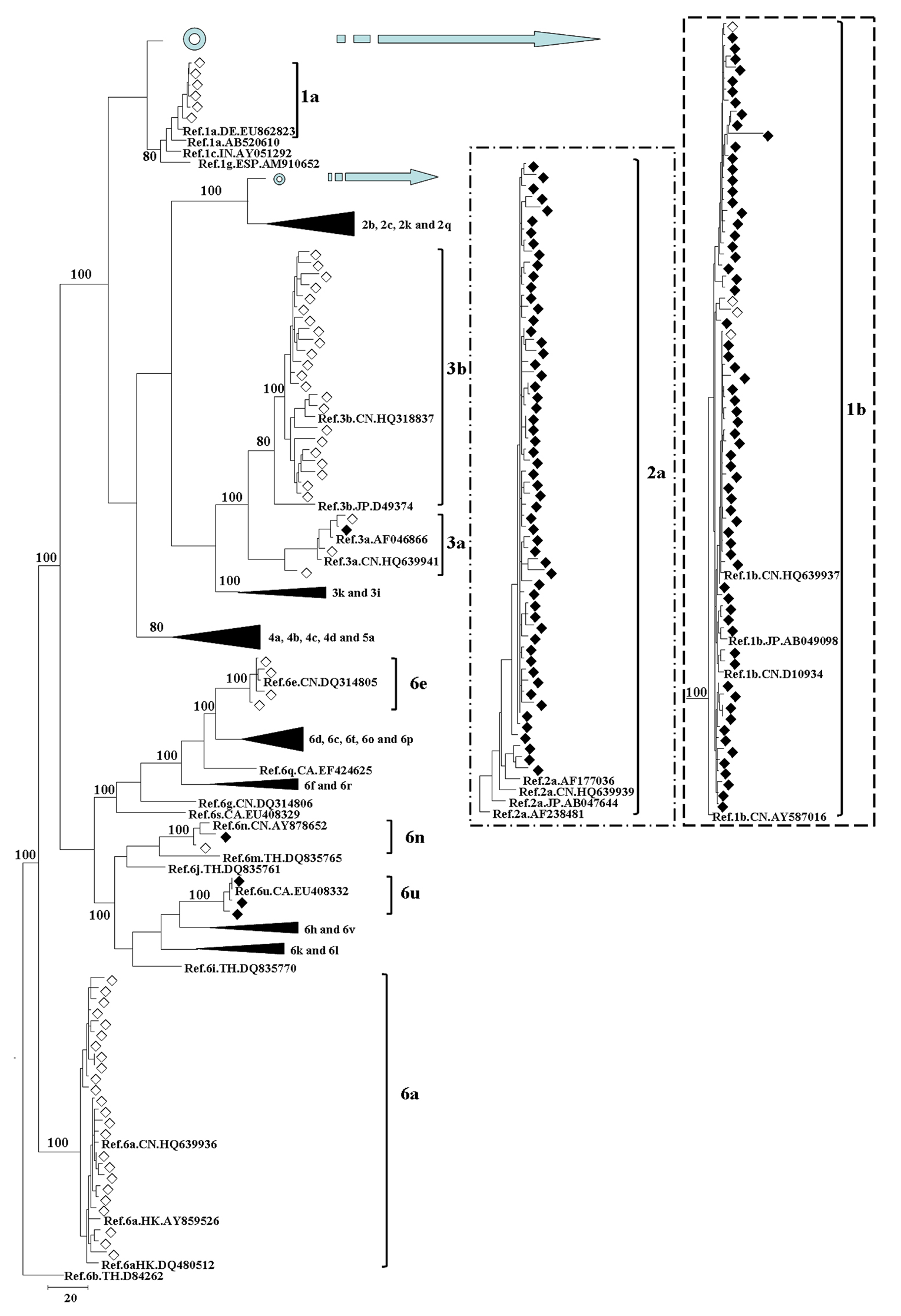

Supplement: Figure S2 — MPT for the HCV C/E2 region sequence obtained from HIV/HCV co-infected patients in Henan and Guangxi. The sequences correspond to nucleotides 915-1,835 in HCV H77 genome (NC_004102). This dataset included sequences from 241 HCV specimens. The dataset had an aligned length of 945 characters in the dataset, of which, 200 characters are constant, 70 are variable and parsimony-uninformative, and 675 are parsimony-informative. Maximum Parsimony analysis yielded 100 equally parsimonious trees (TL = 2911, CI = 0.175, RI = 0.823, RC = 0.144, HI = 0.825). Parsimony bootstrap proportions higher than 70% were indicated along branches. See Figure 1 for reference sequences naming details and signing scheme. (TIF) [file pone.0050343.s002.tif]

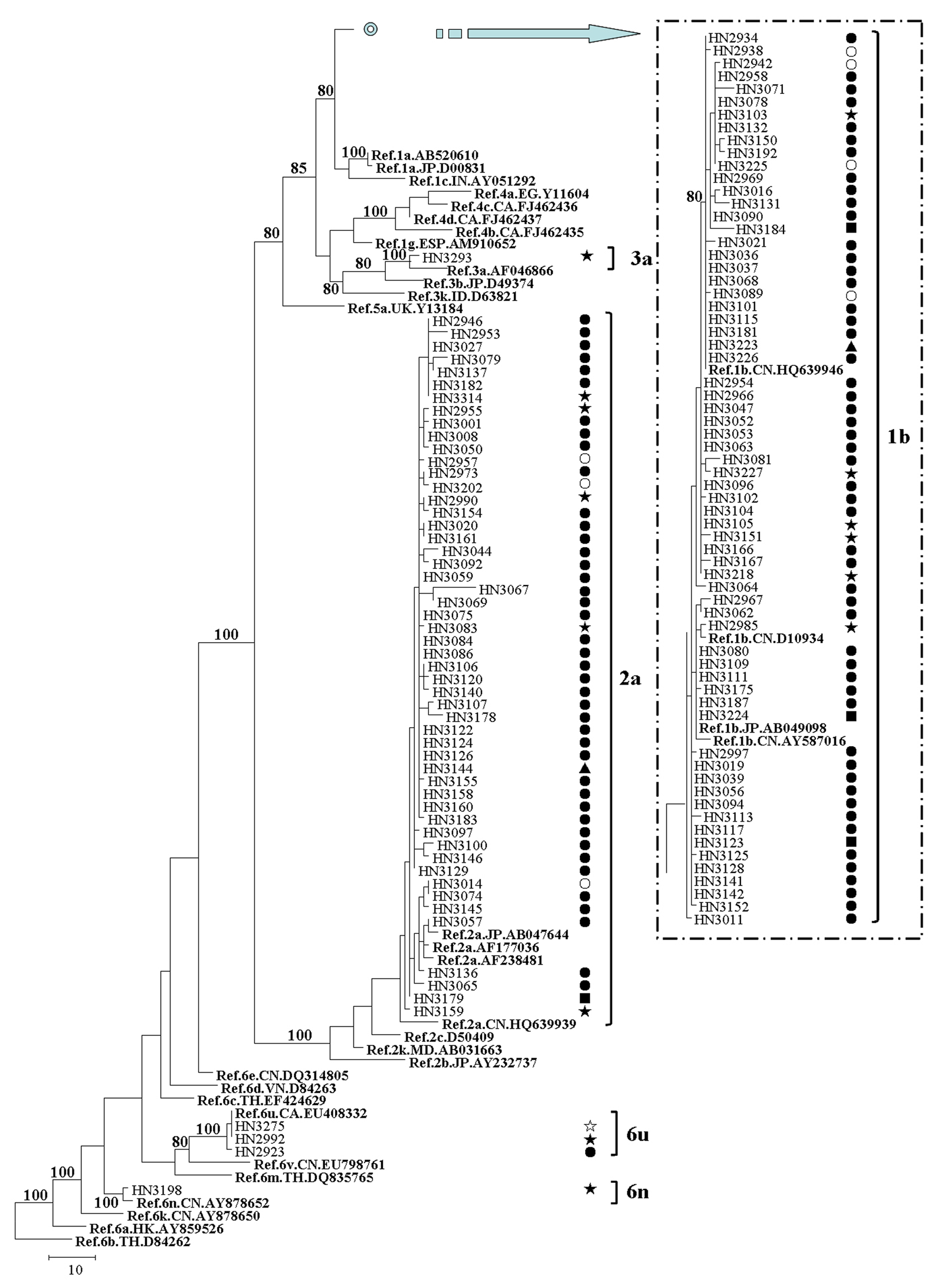

Supplement: Figure S3 — MPT for the HCV 5′NCR region sequence obtained from HIV/HCV co-infected patients in Henan. The sequences correspond to nucleotides 91-681 in HCV H77 genome (NC_004102). This dataset included sequences from 156 HCV specimens. The dataset had an aligned length of 594 characters in the dataset, of which, 373 characters are constant, 60 are variable and parsimony-uninformative, and 161 are parsimony-informative. Maximum Parsimony analysis yielded 9 equally parsimonious trees (TL = 727, CI = 0.305, RI = 0.838, RC = 0.256, HI = 0.695). Parsimony bootstrap proportions higher than 70% were indicated along branches. See Figure 1 and Figure 2 for reference sequences naming details and signing scheme. (TIF) [file pone.0050343.s003.tif]

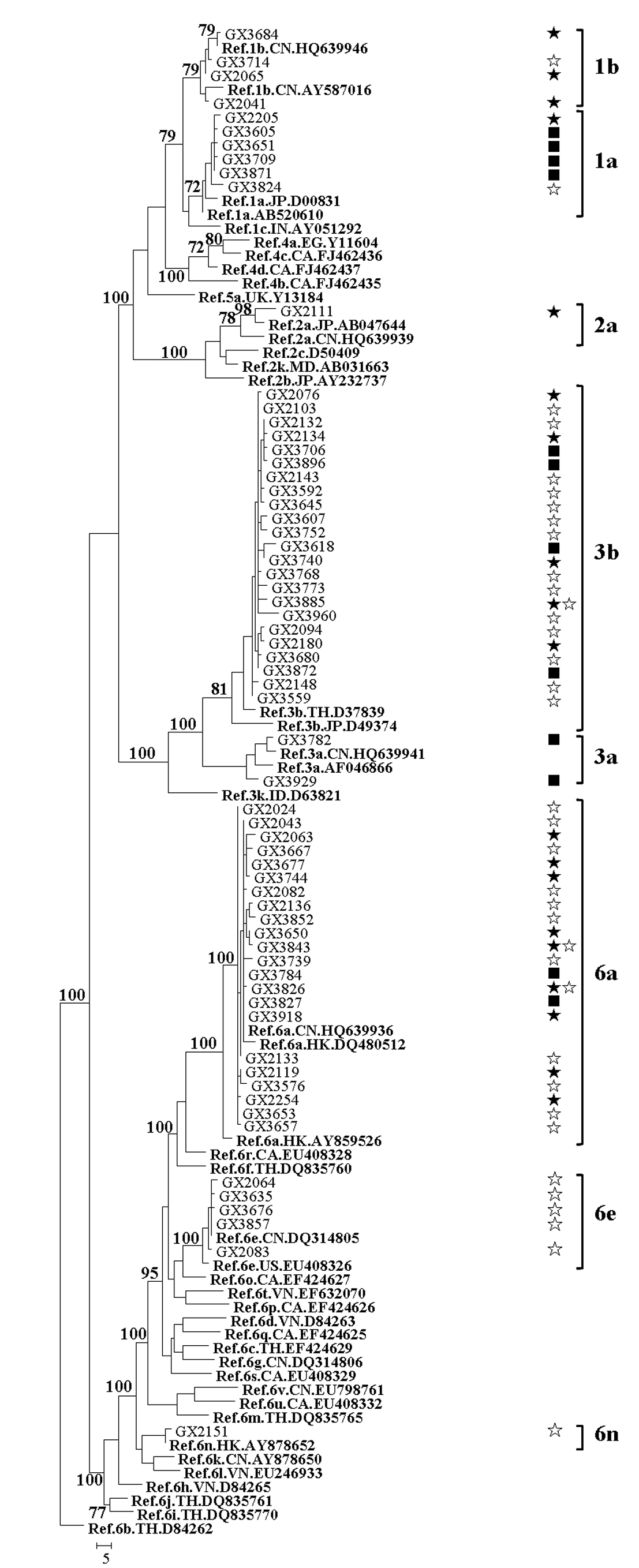

Supplement: Figure S4 — MPT for the HCV 5′NCR region sequence obtained from HIV/HCV co-infected patients in Guangxi. The sequences correspond to nucleotides 91-681 in HCV H77 genome (NC_004102). This dataset included sequences from 109 HCV specimens. The dataset had an aligned length of 594 characters in the dataset, of which, 363 characters are constant, 71 are variable and parsimony-uninformative, and 160 are parsimony-informative. Maximum Parsimony analysis yielded 25 equally parsimonious trees (TL = 924, CI = 0.289, RI = 0.774, RC = 0.224, HI = 0.711). Parsimony bootstrap proportions higher than 70% were indicated along branches. See Figure 1 and Figure 2 for reference sequences naming details and signing scheme. (TIF) [file pone.0050343.s004.tif]

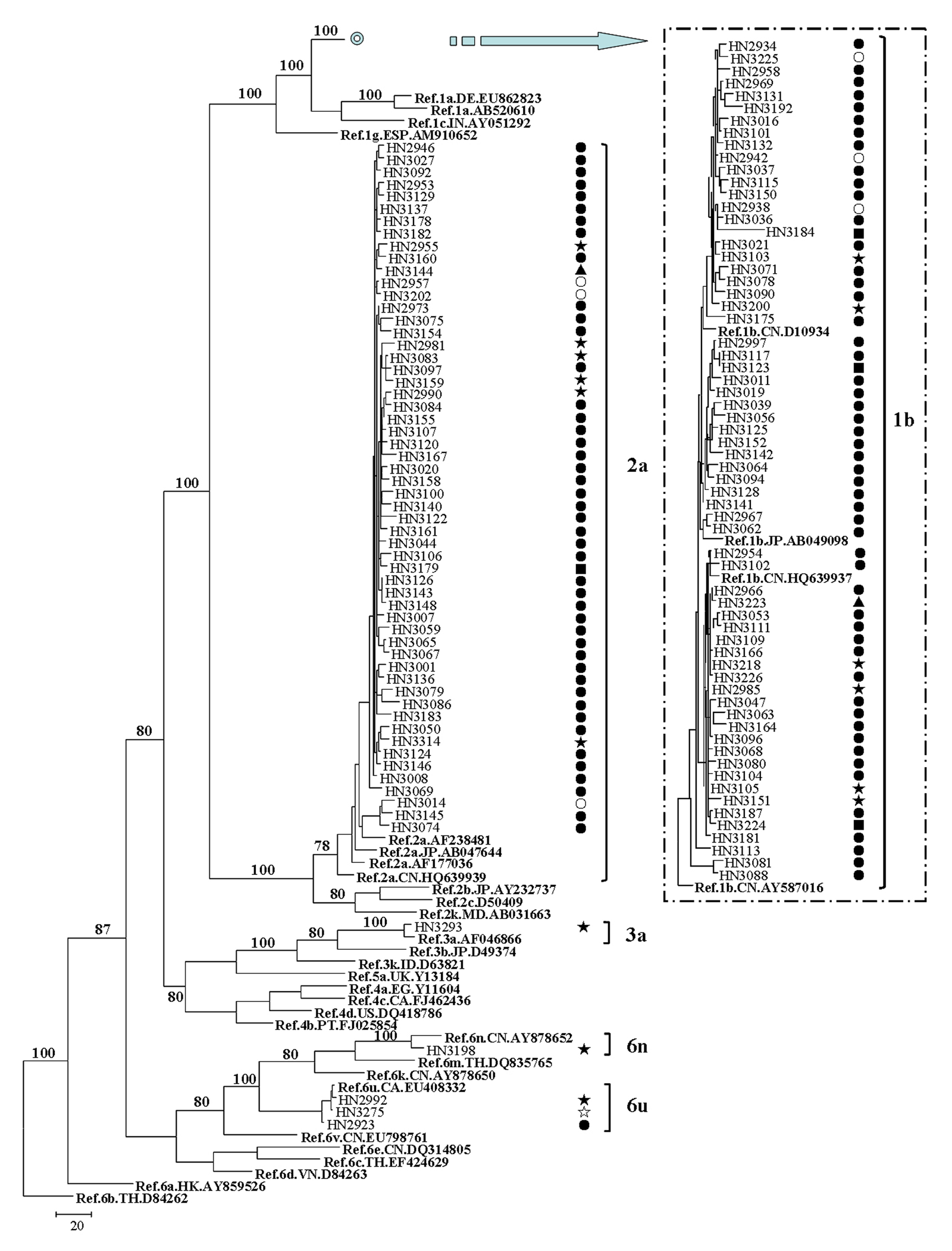

Supplement: Figure S5 — MPT for the HCV C/E2 region sequence obtained from HIV/HCV co-infected patients in Henan. The sequences correspond to nucleotides 915-1,835 in HCV H77 genome (NC_004102). This dataset included sequences from 159 HCV specimens. The dataset had an aligned length of 945 characters in the dataset, of which, 221 characters are constant, 65 are variable and parsimony-uninformative, and 659 are parsimony-informative. Maximum Parsimony analysis yielded 28 equally parsimonious trees (TL = 2546, CI = 0.231, RI = 0.816, RC = 0.188, HI = 0.769). Parsimony bootstrap proportions higher than 70% were indicated along branches. See Figure 1 and Figure 2 for reference sequences naming details and signing scheme. (TIF) [file pone.0050343.s005.tif]

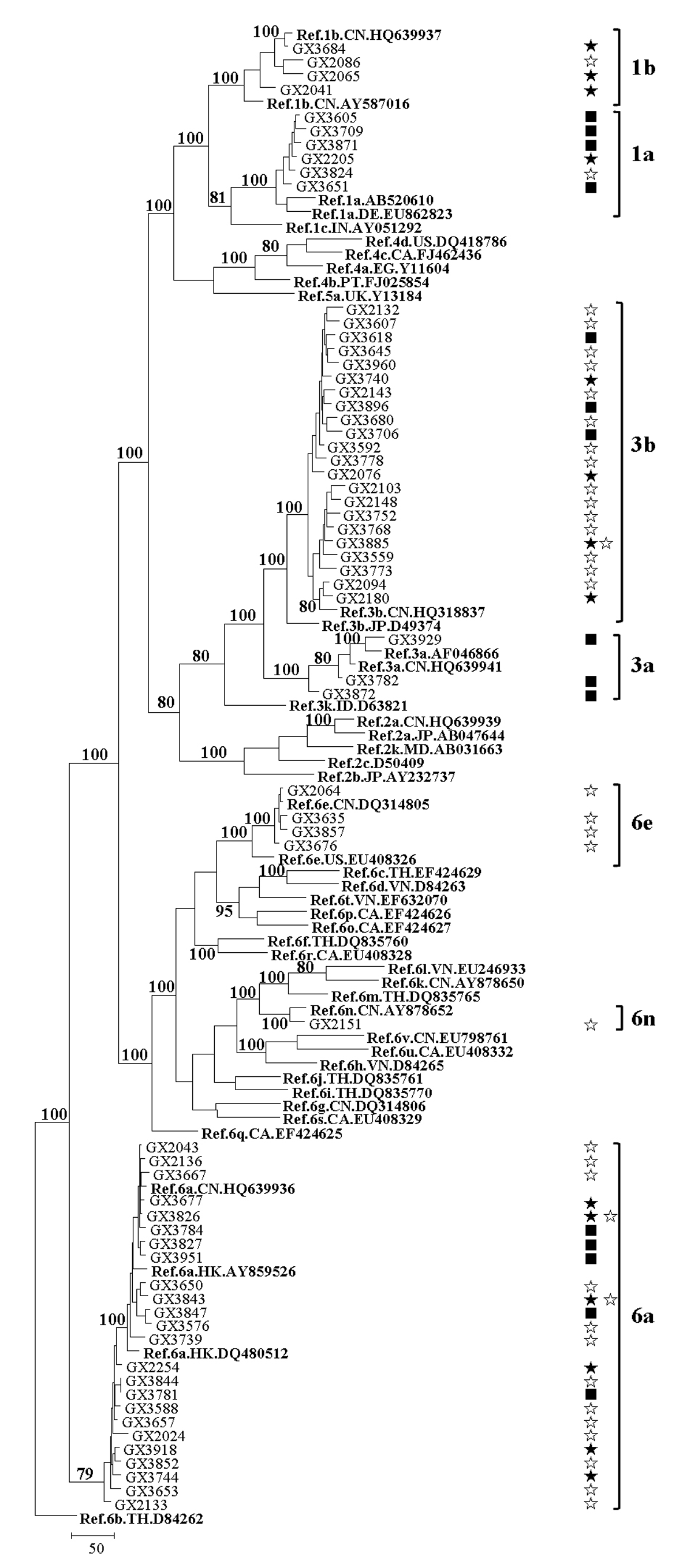

Supplement: Figure S6 — MPT for the HCV C/E2 region sequence obtained from HIV/HCV co-infected patients in Guangxi. The sequences correspond to nucleotides 915-1,835 in HCV H77 genome (NC_004102). This dataset included sequences from 109 HCV specimens. The dataset had an aligned length of 935 characters in the dataset, of which, 217 characters are constant, 51 are variable and parsimony-uninformative, and 667 are parsimony-informative. Maximum Parsimony analysis yielded 28 equally parsimonious trees (TL = 4759, CI = 0.191, RI = 0.693, RC = 0.132, HI = 0.809). Parsimony bootstrap proportions higher than 70% were indicated along branches. See Figure 1 and Figure 2 for reference sequences naming details and signing scheme. (TIF) [file pone.0050343.s006.tif]
